# The Impact of obesity and diabetes mellitus on pancreatic cancer: Molecular mechanisms and clinical perspectives

**DOI:** 10.1111/jcmm.15413

**Published:** 2020-05-26

**Authors:** Bao Quoc Lam, Sushant K. Shrivastava, Anju Shrivastava, Sharmila Shankar, Rakesh K. Srivastava

**Affiliations:** ^1^ Stanley S. Scott Cancer Center Louisiana State University Health Sciences Center New Orleans LA USA; ^2^ Department of Pharmaceutics Indian Institute of Technology Banaras Hindu University Varanasi UP India; ^3^ Department of Oncology St. Joseph's Hospital and Medical Center Phoenix AZ USA; ^4^ Department of Genetics Louisiana State University Health Sciences Center New Orleans LA USA; ^5^ Southeast Louisiana Veterans Health Care System New Orleans LA USA

**Keywords:** alcoholism, diabetes mellitus, obesity, pancreatic cancers, prevention

## Abstract

The incidence of obesity and type 2 diabetes (T2DM) in the Western world has increased dramatically during the recent decades. According to the American Cancer Society, pancreatic cancer (PC) is the fourth leading cause of cancer‐related death in the United States. The relationship among obesity, T2DM and PC is complex. Due to increase in obesity, diabetes, alcohol consumption and sedentary lifestyle, the mortality due to PC is expected to rise significantly by year 2040. The underlying mechanisms by which diabetes and obesity contribute to pancreatic tumorigenesis are not well understood. Furthermore, metabolism and microenvironment within the pancreas can also modulate pancreatic carcinogenesis. The risk of PC on a population level may be reduced by modifiable lifestyle risk factors. In this review, the interactions of diabetes and obesity to PC development were summarized, and novel strategies for the prevention and treatment of diabetes and PC were discussed.

## 1 INTRODUCTION

**Figure 1:**
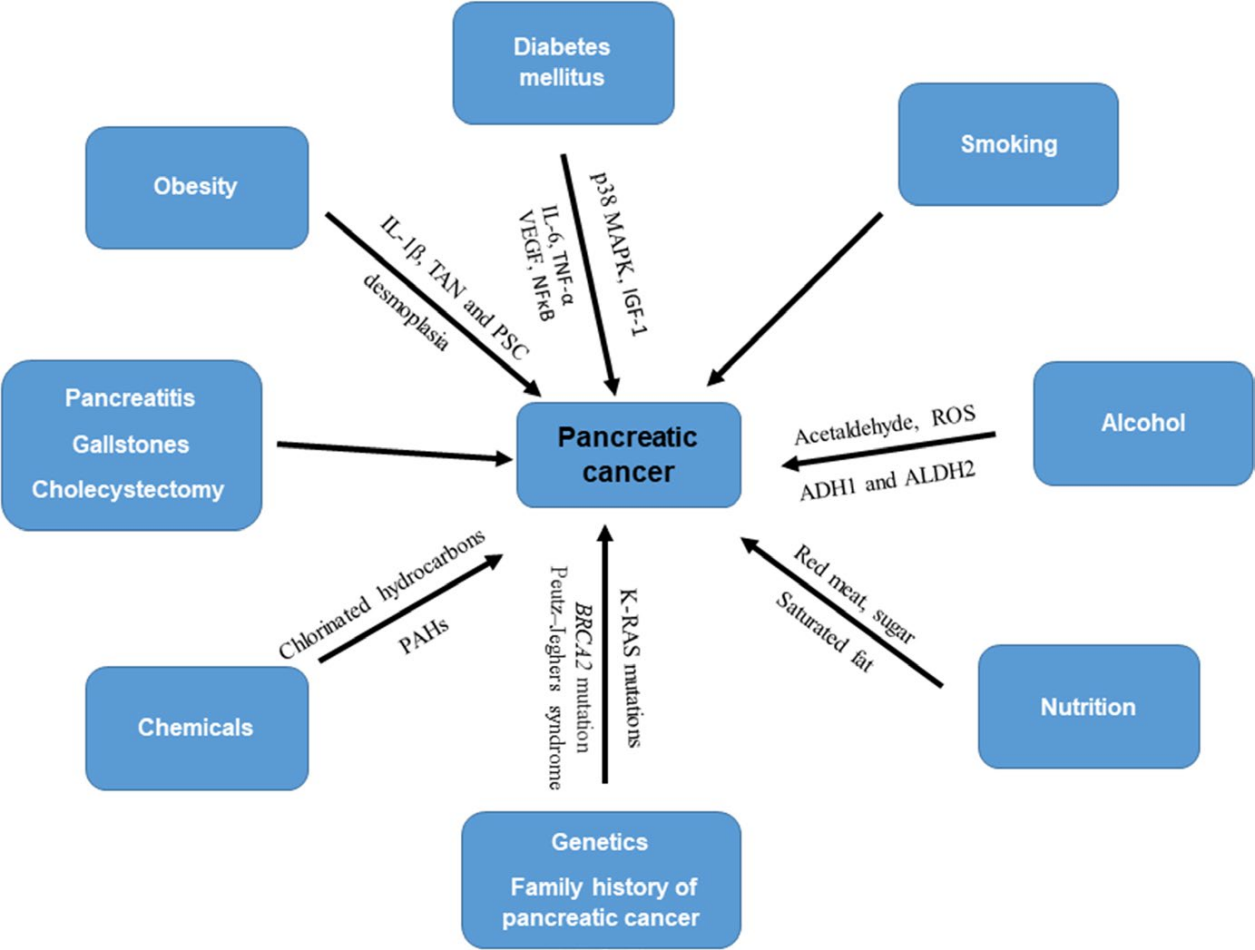
Schema of risk factors of pancreatic cancer. Pancreatic cancer (PC) can be induced by several factors. Smoking is known as the strong carcinogen for PC. Obesity and T2DM synergistically act to induce the development of PC. Other factors such as alcohol abuse, pancreatitis, nutrition, genetics, family history and chemical carcinogen can contribute to the development of PC

Pancreatic cancer (PC) is one of the ten most common cancers in human. Most of the cases are pancreatic exocrine cancer, only 1%‐2% of cases of PC are neuroendocrine tumours. According to the American Cancer Society, the incidence of PC was 53 770 in 2019, with a concomitant mortality of 45 750 (23 800 men and 21 950 women).^1^ It is the fourth cause of cancer‐related death in both men and women in the United States each year.^1^ In the United States, the number of new cases of PC was 12.4 per 100 000 men and women per year based on 2009‐2013 cases. In spite of massive effort on diagnosis and treatment, the 5‐year survival rate has been increased to a mere 8%.^1^ By 2030, the number of deaths from PC will surpass breast, prostate and colon cancer and become the second leading cause of cancer‐related death in the United States.^2^ Due to unclear symptoms and no screening recommendations, a vast majority of PC patients are diagnosed at late stages, with already advanced disease and no opportunity for surgical intervention. The risk factors for PC include tobacco products, obesity, diabetes, chronic pancreatitis, alcohol abuse, malnutrition, hereditary conditions and family history (Figure 1).^3^, ^4^ Diabetes mellitus (DM), or impaired glucose tolerance, is concurrently present in 50%‐80% of patients with PC. DM is a known risk factor for PC,^5^, ^6^ and new‐onset DM could be an early manifestation of PC,^7^ resulting from insulin resistance induced by a paraneoplastic syndrome ^8^ or pancreatic β‐cell dysfunction.^9^, ^10^ In addition, it has been demonstrated that moderate alcohol consumption had insignificant impact, while high alcohol intake was associated with an increased risk of PC.^11^, ^12^ Although the effects of DM and alcohol abuse on the development of PC have been studied for the last few decades, their molecular mechanisms of action are not well understood. We conducted this review to update and summarize the mechanisms of association among diabetes mellitus, obesity, alcoholism, other factors and cancerous pancreas. In addition, prevention and treatment strategies are also critically discussed in this review paper.

## 2 DIABETES MELLITUS (DM), A RISK FACTOR OF PANCREATIC CANCER

There are three major types of diabetes mellitus (DM): type 1, type 2 and gestational diabetes. Type 1 diabetes (T1DM) may account for about 5% of all cases of diabetes. Type 2 diabetes account for about 90% to 95% of diabetes. In obese individuals with euglycaemia, peripheral insulin resistance is present but compensated by increased insulin secretion.^13^, ^14^ Increase in insulin resistance and β‐cell dysfunction, and reduction in β‐cell mass occur over time, finally leading to T2DM.^13^, ^14^ Unlike T2DM, studies conducted on small on number of patients have not shown that T1DM is risk factor of PC.^15^, ^16^ Patients with T2DM possess a threefold risk of developing PC.^17^ Consistently, duration of the diabetes increases the risk of developing PC in a prospective study with hazard risk 2.0 in both men and women.^18^ Although HbA1C is used as a marker of metabolic control, some studies have suggested its use as a predictor and prognostic factor in PC. Moreover, hyperglycaemia in diabetic patients promotes the growth of solid tumours and metastasis to distant organs in cancers.^19^

## 3 POTENTIAL MECHANISMS OF DIABETES ON THE DEVELOPMENT OF PANCREATIC CANCER

**Figure 2:**
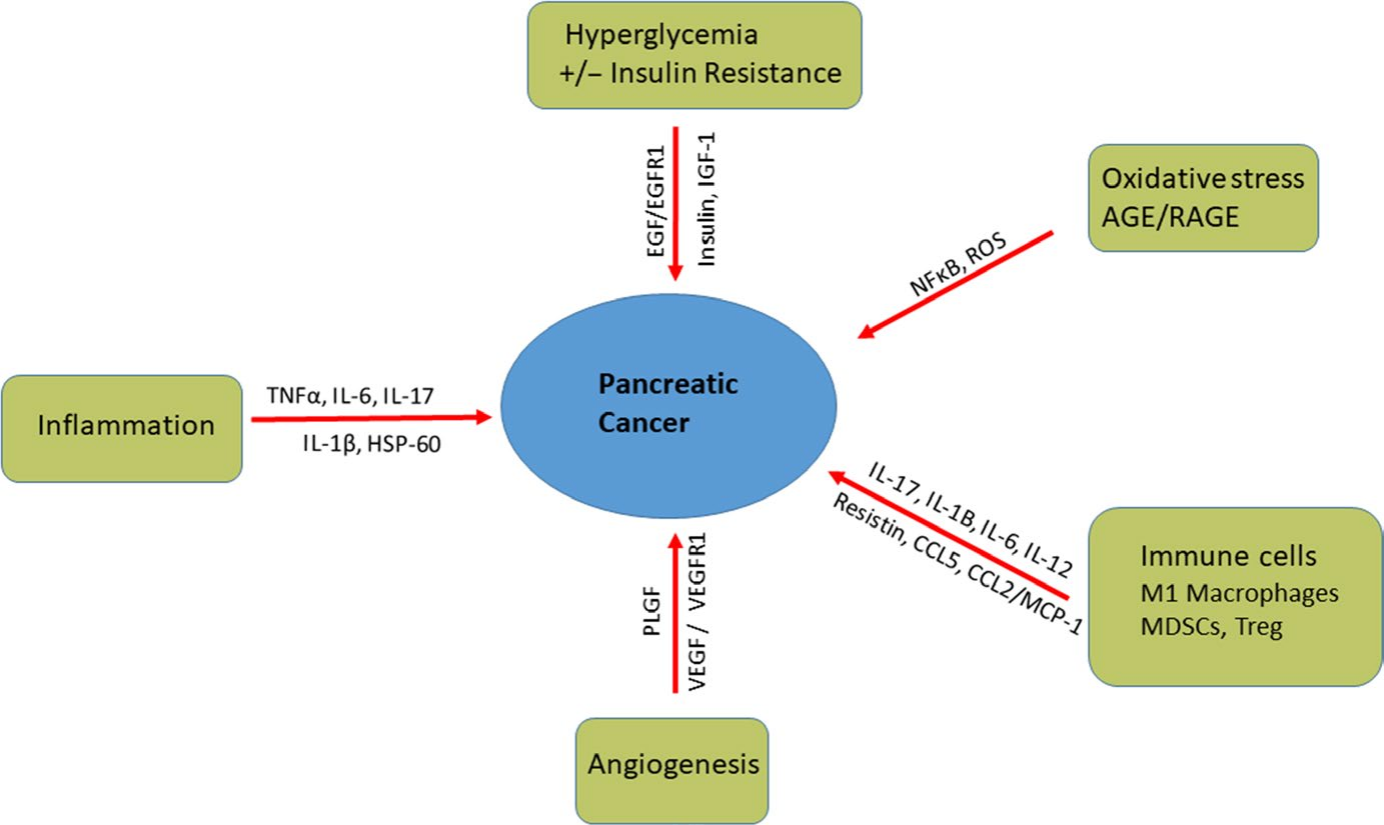
Pathogenesis of pancreatic cancer. Pancreatic cancer can be initiated and accelerated by several factors. Hyperglycaemia with or without insulin resistance is a risk factors of PC. Inflammation, oxidative stress and certain immune cells can initiate the development of PC. Angiogenesis can enhance the development of PC

The pathogenesis of PC in DM or hyperglycaemia has been substantially studied (Figure 2). High glucose‐activated p38 MAPK induced the proliferation and invasion of PC cells.^20^ In response to cellular stress and inflammatory conditions, P38 MAPK is activated.^21^ In addition, the proliferation, apoptosis and metastasis can also be regulated by the p38 MAPK.^22^, ^23^ P38 MAPK enhanced inflammatory cytokine (IL‐6)‐ and VEGF‐mediated paracrine effects, resulting in PC cell growth and development.^24^ Moreover, proliferation and invasiveness in PC cells were promoted by high glucose via RET [proto‐oncogene encodes a receptor tyrosine kinase for members of the glial cell line‐derived neurotrophic factor (GDNF) family of extracellular signalling molecules].^25^ DM stimulates PC growth, epithelial‐mesenchymal transition and metastasis through generation of inflammatory cytokines (IL‐6 and TNFα) and activation of kinases (p38 MAPK and NFκB).^20^ NFкB is not only activated in cancer cells, but also in immune cells where it regulates the production of inflammatory cytokines (IL‐6, IL‐8, IL‐1β and TNFα) triggering PC cell growth.

In type 2 DM (T2DM), elevated insulin levels result in increased bioavailability of insulin‐like growth factor‐1 (IGF‐1) by reducing hepatic production of IGF‐binding proteins (IGFBPs) and PC cells highly express receptors of high‐affinity insulin and IGF‐1.^26^, ^27^ The interaction of insulin, IGF‐1 and their receptors plausibly induces more activated PC cells. Insulin regulates glucose uptakes in target tissues and also acts as a mitogen for PC cells.^28^, ^29^ IGF‐1 besides its mitogenic effects, induces angiogenesis and increases EMT and metastasis, and blocks apoptosis, thereby enhancing PC growth.^30^ Higher insulin and IGF‐1 levels in T2DM patients were associated with larger pancreas tumours than in non‐diabetic controls.^31^, ^32^ The prevalence of PC in T1DM is lower than in T2DM. It is not clear whether hyperglycaemia alone or the combination of hyperglycaemia with insulin resistance in T2DM act as an initiating factor for PC. Hyperglycaemia has been shown to accelerate the development of PC by providing glucose as a fuel to cancer cells. Some other studies have indicated hyperglycaemia enhanced proliferation via the induction of EGF/EGFR,^33^ local invasiveness and metastatic potential in PC via neural and perineural environment.^34^ Hyperglycaemia has been shown to induce endothelial dysfunction and neo‐angiogenesis,^35^ resulting in endothelial discontinuities, large blood‐filled spaces and decreased vessel density via angiopoietin‐2.^36^

In another aspect, receptors of advanced glycation end products (RAGE), another cancer‐related factor, are constitutively expressed on the epithelial, immune, endothelial and vascular smooth muscle cells.^37^ There is still a paradox about the role of AGE/RAGE in cancerous pancreas. The RAGE has been shown to enhance pancreatic carcinogenesis by amplifying IL‐6‐induced autophagic translocation of STAT3 to the mitochondria in mice.^38^ However, human studies failed to show any association between AGE/RAGE and PC risk.^39^ This controversy is probably explained by the fact that AGE/RAGE could be only functional in the earlier stages of pancreatic carcinogenesis; and smoking, another risk factor, in the latter stages could affect to the role of AGE/RAGE in tumorigenesis. Alternatively, the disparity is simply due to the difference between human and mouse models. However, other studies showed a positive correlationship between the AGE/RAGE interaction and development of pancreatic and gastric cancers, and melanoma.^40^, ^41^, ^42^ AGE/RAGE induces pro‐inflammatory cytokines and generates oxidative stress and reactive oxygen species, resulting in activation of NFкB and its target genes.^43^

Thrombospondin‐1 (TSP‐1), adhesive glycoprotein, mediates cell‐to‐cell and cell‐to‐matrix interactions and play crucial roles in platelet aggregation. TSP‐1 has been described as anti‐carcinogenic due to its potent anti‐angiogenic properties.^44^ In addition, a significant reduction in TSP‐1 was reported up to 24 months prior to diagnosis of PC with DM compared to non‐cancer DM.^45^ These findings suggest that TSP‐1 could be a novel target in lowering the risk of PC. Aside from that, miRNAs are being evaluated as novel therapeutic targets and biomarkers for PC. A combination of serum miRNAs (miR‐483‐5p, miR‐19a, miR‐29a, miR‐20a, miR‐24, miR‐25) is potential biomarker for the diagnosis and discrimination of PC‐DM from non‐cancer new‐onset DM.^46^, ^47^ Future studies are needed to validate the function of these miRNA in PC.

The bromodomain and extraterminal domain (BET) proteins are a bromodomain subfamily that includes BRD2, BRD3, BRD4 and BRDT.^48^, ^49^, ^50^, ^51^, ^52^ The BET proteins utilize bromodomains to interact with acetylated histone tails and mediate downstream functions, such as histone acetylation recognition, chromatin remodelling and transcription regulation. They have been implicated to play roles in inflammation, apoptosis, cell proliferation and cell cycle and cancer.^50^, ^53^ Bromodomain containing protein 4 (BRD4) is a coactivator of NFkB, enhances the transcription of NFkB‐dependent pro‐inflammatory cytokine genes.^54^ In addition to activating NFkB, BET proteins also regulate STAT signalling pathway.^55^ The inhibition of BET protein attenuates cytokine production (such as IL‐6, IL‐17 and IL‐1β), which have been implicated in diabetogenic inflammation.^56^, ^57^ As a linkage of diabetes and inflammation, Hsp60, a mitochondrial stress protein, regulate immune inflammation by acting as a ligand for innate immune receptors and adaptive immune receptors.^58^

Immune cells, including macrophages, B cells and T cells, play key roles in the progression of T2DM. They contribute to cytokine driven diabetogenic inflammation and the development of T2DM.^59^, ^60^, ^61^ The most common pro‐inflammatory T‐cell subsets implicated in diabetogenic inflammation are Th1 cells, Th17 cells and CD8+T cells.^62^, ^63^, ^64^, ^65^ It was shown that T cells from humans with T2DM only produce levels of pathogenic IL‐17 in the presence of B cells^66^ which was found in visceral adipose tissue of obese people.^67^ IL‐17 produced by γδ T cells drives neutrophil polarization into a myeloid‐derived suppressor cell (MDSC)‐like phenotype, resulting in inhibition of cytotoxic CD8+T cells and promotion of metastasis.^68^ CD4+T cells tend to polarize to pro‐inflammatory Th1 and Th17 cells in peripheral blood and adipose tissue of T2DM patients. In contrast, polarization of anti‐inflammatory Th2 cells was decreased. M1 pro‐inflammatory macrophages induced the secretion of IL‐1β, IL‐6, IL‐12, TNF‐α, CCL5 and CCL2 in adipose tissue during obesity.^69^, ^70^ During islet inflammation, macrophage transformation shifts towards the pro‐inflammatory activated M1‐like phenotype, which contributes to β cell dysfunction in T2DM.^71^, ^72^ Consumption of foods high in saturated fatty acids (palmitate) is known as one of the risk factors of T2DM. There was palmitate‐dependent recruitment of CD11b+Ly‐6C+classically activated M1‐like monocytes/macrophages to the islets.^73^ TLR4 in islet cells and Myd88 were required for M1‐like macrophage recruitment, leading the β cell dysfunction via secretion of IL‐1β.^73^

## 4 OBESITY AND PANCREATIC CANCER

The prevalence of obesity is on the rise worldwide and is considered a serious pandemic today. It is now well accepted that the obesity is one of the leading risk factors for PC.^3^, ^74^, ^75^, ^76^ Although obese people possess an increased risk of developing PC, patients with increased pancreatic fat have a poorer outcome than those who develop cancer in a lean pancreas.^77^ The mechanisms by which obesity mediates the risk for PC are not well understood. Studies have suggested the involvement of inflammation and hormonal imbalance. Emerging evidence demonstrates that the increase of certain hormones in obese patients, such as insulin, adipokines and resistin, and oxidative stress are responsible for the initiation and progression of PC.^78^, ^79^ Adipocytes in obesity secreted high IL‐1β, recruiting tumour‐associated neutrophils (TAN) which induces activation of pancreatic stellate cells (PSC). IL‐1β, TAN and PSC induced the aggravation of desmoplasia which is modulated by angiotensin‐II type‐1 receptor, and promoted PC progression.^80^ Obesity has been found to be associated with increased systemic levels of placental growth factor (PLGF).^81^, ^82^ PLGF/VEGFR‐1 system modulates angiogenesis and promotes tumour‐associated macrophage (TAM) recruitment and activity in PC.^83^ Therefore, targeting PLGF/VEGFR‐1 signalling may be an attractive strategy to reprogramme the tumour immune microenvironment and inhibit obesity‐induced acceleration of PC progression.^84^

Recent studies have shown a link between obesity and obesity‐related PC through adipokines.^85^, ^86^ Epidemiologic studies reported low adiponectin levels in human obesity and have been associated with increased PC risk. As adiponectin can suppress PC growth by inhibiting the β‐Catenin signalling pathway,^87^ the activation of adiponectin signalling could be a novel therapeutic strategy for obesity‐related PC.

Obesity is the leading risk factor associated with the development of T2DM. Obesity causes the activation of multiple inflammatory signalling pathways. Some of these pathways are associated with adipose tissue hypoxia,^88^, ^89^ leptin secretion and unfolded protein responses that are activated by endoplasmic reticulum stress.^90^ Secretion of inflammatory cytokines (eg TNFα, IL‐6) and increased lipolysis can also occur during obesity‐related complications.^91^ Visceral adipose tissue (VAT) is one of the major sites for the inflammation that is associated with DM.^59^, ^92^ Immune cells infiltrate VAT, resulting in low‐grade chronic inflammation and secretion of pro‐inflammatory cytokines that can induce insulin resistance, both locally and systemically.^93^

The abdominal adiposity is one of the modifiable risk factors for PC onset, suggesting weight loss could manifest an effective preventive measure. Lifestyle modifications (eg exercise and healthy diets) for the purpose of reducing obesity could also reduce PC rates. In cases when these measures alone were found to be ineffective, bariatric (metabolic) surgery was a useful alternative.^86^, ^94^, ^95^ Bariatric surgery in extremely obese patients may produce many health benefits along with weight loss, consequently reducing the chances of PC, especially when additionally incorporating healthy lifestyle habits.

## 5 GENETIC MUTATIONS IN PANCREATIC CANCER

Genome‐wide association studies have shown that the pancreatic development genes, HNF1A, HNF1B, HNF4G, PDX1 and NR5A2 contributed to the significant association of PC.^85^, ^96^, ^97^ HNF1A and HNF1B are also associated with T2DM.^85^, ^96^ In addition, the glucokinase regulator single‐nucleotide polymorphism (SNP) rs780094 with CC genotype was associated with PC risk in T2DM patients.^98^ The DM risk alleles of two SNPs, FTO rs8050136 and MTNR1B rs1387153, showed slight but significant associations with increased PC risk,^99^, ^100^ supporting with the notion that DM or obesity (which is influenced by the FTO locus)^101^ increases PC risk.^102^ The KRAS mutations are among the earliest genetic alterations which have been associated with the initiation of PC.^4^, ^103^, ^104^

Apart from that, the role of *BRCA2* alterations has been used in early diagnosis and prediction of familial PC patients.^105^ *BRCA2* mutation was a late event in sporadic pancreatic tumorigenesis ^106^ preceded by *KRAS* mutation (G12D) or loss of TP53.^107^ *PALB2* was found to be the second most commonly mutated gene for hereditary PC.^108^ Interestingly, the most commonly mutated gene was BRCA2, whose protein product was capable of binding with the *PALB2* protein.^109^ The germline mutations of STK11 (a tumour suppressor gene) are associated with Peutz–Jeghers syndrome which increases the risk of PC.^110^ Moreover, mutations in STK11 gene have also been reported in sporadic PC.^111^, ^112^

## 6 MICROENVIRONMENT, IMMUNE CELLS AND PANCREATIC CANCER

Microenvironment plays a major role in cancer initiation, progression and metastasis.^113^ Stroma consists of various cell types including fibroblasts and immune cells, and the extracellular matrix (ECM) secreted by the cellular components. In mouse model of PC, targeting the hyaluronan in the ECM of stroma relieved the pressure on the blood vessels resulting in enhancing the survival of tumour‐bearing mice.^114^ In contrast, PEGylated hyaluronidase failed to improve the survival of patients.^115^ Signalling pathways in stroma environment play significant roles in chemotherapy resistance. For example, inhibition of the Sonic Hedgehog (Shh) pathway depleted tumour‐associated stromal tissue, increased micro‐vessel density and drug delivery in animals^116^ but failed to offer the benefit to patients. The tumour‐associated stroma (TAS) is responsible for deposition of collagen and various ECM components that stimulate cancer cell proliferation and angiogenesis.^117^ MyD88‐dependent TAS responded to PC cell–secreted factors resulting in loss of Th1 function and inhibition of cytotoxicity by the CD8+T cells.^117^, ^118^ Pancreatic cancer cells express prostate stem cell antigen (PSCA). Immunosuppressive function of IL‐4 in microenvironment was investigated by using the fusion of IL‐4 receptor exodomain with immunostimulatory IL‐7 receptor endodomain in PSCA‐targeting T cells, resulting in potent and sustained anti‐tumour effects.^119^ Shh/Gli signalling pathway plays a significant role in both epithelial and stromal cells of PDAC tumours.^120^ In PDAC, the inhibition of bromodomain and extraterminal domain (BET) protein resulted in down‐regulation of Shh.^120^ Shh ablation resulted in diminished stroma formation, reduced survival due to formation of more aggressive, dedifferentiated tumours and increased metastases.^121^ Inconsistently, stromal depletion resulted in decreased survival with similarly aggressive tumours.^122^ The increased tumour vascularity with stromal depletion correlated with disease progression but also increased responsiveness to anti‐angiogenic agents. Elevated pro‐angiogenic vascular endothelial growth factor A (VEGF‐A) levels in patients have been found to correlate with increased vascular density of PDAC and greater disease progression.

Accumulation of hyaluronan/ hyaluronic acids (HA) promotes tumour growth in mice and correlates with poor prognosis in patients with PDAC. Inhibiting HA signalling or depleting HA levels in tumour stroma could represent a promising therapeutic strategy against PDAC. Production of HA was used as the primary determinant of elevated intratumoural pressures. In PDAC, targeting the HA in the ECM of stroma significantly reduced the pressure on the blood vessels and enhanced the survival of tumour‐bearing mice.^114^ Indeed, treatment with human recombinant hyaluronidase (PEGPH20) relieved intratumoural pressures, increase in tumour vascular perfusion and gemcitabine delivery, improved survival and decreased the metastatic burden.^123^

Regulatory T (Treg) cells, myeloid‐derived suppressor cells (MDSCs) and macrophages appears early in PDAC development. T helper 17 (TH17) with few CD8+lymphocytes were also found in the microenvironment of the KRAS PDAC model.^124^ Depletion of carcinoma‐associated fibroblasts (CAFs) that express fibroblast activation protein (FAP) resulted in an increased anti‐tumour cytotoxic T‐cell‐mediated inhibition of PDAC.^125^ Furthermore, blocking the activity of CAF‐secreted cytokine CXCL12 induced rapid T‐cell accumulation in tumours and acted synergistically with PD‐L1 to greatly deplete cancer cell.^125^ Analysis of resected tumour specimens demonstrated that that intratumoural levels of IL‐8, IL‐1β and TNFα were associated with larger tumours and poor differentiation.^126^ Inflammation within the pancreatic tumour microenvironment has been linked to tumour progression and chemo‐resistance through IL‐6, Toll‐like receptor, NFkB and TGF‐β signalling pathways.^127^ In Kras^G12D^ mouse model of PC, we have demonstrated that Th17, PMN‐MDSC, IL‐6 and IL‐8 (Th2 type) cells were up‐regulated, whereas CTL, NKT, γδT, NK and IFNϒ (Th1 type) cells were suppressed.^128^ These studies suggest that microenvironment plays a significant role in the development of PC.

## 7 NUTRITION AND PANCREATIC CANCER

Malnutrition and consumption of unhealthy diets have been associated with incidence of PC; however, the results have been inconsistent.^4^, ^129^ The Western diet, containing high red meat, refined grains and sugar‐sweetened beverages, has been associated with increased levels of systemic inflammatory markers ^130^ and increased risk of PC.^4^ In contrast, the Mediterranean‐style diet, containing plant foods, whole grains and fish, has been associated with reduced levels of inflammatory markers^131^, ^132^ and reduced risk of PC.^133^ The higher inflammatory scores of diet increased the risk of PC with odds ratio (OR) of 2.54,^134^ confirming a previous study on Italian population in which higher OR was associated with PC risk.^135^ Inflammatory conditions created by unhealthy diet may contribute to pancreatic carcinogenesis by increasing blood levels of inflammatory cytokines (eg IL‐6, IL‐8, IL‐1β, TNF‐α and IFN‐ϒ), which can promote excessive levels of reactive oxygen species.^136^ As a result, these inflammatory conditions cause DNA damage, mutagenesis and, thus lead to the development of PC.^136^ Interesting consumption of red and processed meat was shown to increase risk of PC in men but not in women through unknown mechanism.^137^ The consumption of citrus fruits, vitamin B6 and choline has been inversely correlated with the risk of PC.^138^, ^139^ Intake of N‐3 (ω‐3) polyunsaturated fatty acids and docosahexaenoic acid from fish was associated with a lower risk of PC.^140^ Unsaturated fatty acids was associated with decreased risk of developing PC whereas saturated fatty acids found in dairy products increased the risk of PC.^141^ Like saturated fatty acids, higher consumption of sugar and high‐sugar foods was associated with a greater risk of PC.^142^ The intake of folate also lowered the risk of PC.^143^, ^144^ The folic acid exerts its effects via reducing RhoA activity mediated by activation of the FR/cSrc/p190RhoGAP signalling pathway.^145^ Dietary fat‐induced PC growth and metastasis via endogenous cholecystokinin (CCK).^146^

Purified natural products have shown promising results in cell culture and mouse models of diabetes and PC.^4^ Epigallocatechin‐3‐gallate (EGCG), a polyphenolic compound from green tea, showed anticancer activities in cell culture and mouse xenograft model of PC.^147^, ^148^ Several other natural products such as mangostin, curcumin, resveratrol, silibinin, sulforaphane, anthothecol, berberine and embelin have demonstrated anticancer activities against PC.^4^, ^128^, ^149^, ^150^, ^151^, ^152^, ^153^, ^154^, ^155^, ^156^, ^157^, ^158^, ^159^ These agents inhibited PC growth, development and metastasis by generally suppressing NFκB, AKT, Shh, Notch or Wnt pathways.^4^, ^160^ Although effective in mouse models, these agents have not demonstrated satisfactory pharmacokinetic (PK) and pharmacodynamics (PD) profiles in clinical trials. Nanotechnology appears to be promising in improving the PK/PD profiles of natural products in mouse models of PC and diabetes.^4^, ^158^, ^161^, ^162^, ^163^ Clinical trials are needed to assess the therapeutic potential of nanoparticles containing natural products for the treatment and/or prevention of PC and diabetes.

## 8 ANTIHYPERGLYCAEMIC THERAPY AND RISK OF PC

There is some evidence that therapies that increase insulin levels such as exogenous insulin may have potential increase PC risk.^164^ Metformin (T2DM drug) can decreases insulin levels by decreasing insulin resistance and also reduces the risk of pancreatic malignancy with hazard ratio of 0.15‐0.54.^165^ The use of metformin in patients with diabetes and pancreatic malignancy prolonged median survival compared to control (non‐users): 16 vs 11 months.^166^, ^167^, ^168^, ^169^, ^170^ Although an anti‐tumour effect of metformin has been shown in preclinical and epidemiological studies, other studies have not shown a consistent survival benefit from metformin in PC patients with pre‐existing diabetes.^171^, ^172^ Metformin is known to activate the LKB1‐AMPK pathway, a cellular energy stress‐sensing mechanism, and blocks proliferation through inhibition of mTOR which also enhances cell proliferation by activation the insulin signalling pathway.^173^, ^174^ In addition to reducing glucose levels in T2DM, the use of metformin in prevention of PC is very promising and needs further evaluation.

## 9 PREVENTION OF PANCREATIC CANCER

The first step in preventive strategy is avoiding the exposure to the modifiable risk factors such as smoking, drinking, obesity, carcinogenic chemicals and some specific foods (saturated fats). PC protecting foods (polyphenols or flavonoids, folic acid and fish) should be included in daily dietary. Recent studies have recommended the use of polyphenols rich in specific foods (cloves, peppermint, star anise, cocoa powder and so on) as a chemopreventive approach for PC.^175^, ^176^ The health‐related properties of a wide range of dietary constituents show potential biological activities against PC.^177^ Resveratrol, a red wine polyphenol, can directly bind and inhibit leukotriene A4 hydrolase (LTA‐4H) activity and, therefore, induces the production of LTB4^178^ which prevents the development of PC.^154^, ^179^ Most natural products including curcumin inhibit PC growth and metastasis by suppressing NF‐κB and its targets.^179^, ^180^ Therefore, resveratrol and curcumin can be used for preventing PC in high‐risk patients. The preliminary data on resveratrol and curcumin indicated potential benefits but needs confirmation in clinical trials. It is also critical to diagnose and start radical treatment for gallstones, cholecystitis and chronic pancreatitis and set up a PC screening in these high‐risk patients. So far, no tumour‐specific markers with high sensitivity and specificity have existed for PC. The carbohydrate antigen 19‐9 (CA19‐9) as a prognostic marker of PDAC has been used with a limited success. Serum CA19‐9 maker has low specificity but high sensitivity.^181^, ^182^ Using quantitative proteomic analysis, C4b‐binding protein alpha‐chain (C4BPA) was identified as a novel serum biomarker for diagnosis of early stage PDAC.^183^ Therefore, CA19‐9 and C4BPA could be used for screening high‐risk individuals with PC.

## 10 CONCLUSIONS

In spite of enormous research efforts, pancreatic cancer remains a deadly disease with incremental benefits seen with cytotoxic chemotherapy in recent years. Fortunately, the treatment of patients with chemotherapy has nearly doubled median overall survival but still remains very poor (<1 year). Immunotherapy, vaccine and checkpoint inhibition appear to be emerging modalities for PC. Diabetes, obesity and dietary patterns are closely correlated to cancer risk. Thus, avoiding these risks will obviously attenuate the morbidity and mortality of cancers. Furthermore, regular exercise and improving lifestyle can help in reducing obesity and diabetes. In addition, the discovery of new cancer‐related genes is critical for the cancer screening strategy and prevention. Eventually, the better comprehension of pathogenesis and novel therapies should be more extensively studied to lower or eliminate morbidity and mortality of PC.

## CONFLICT OF INTEREST

All the authors confirm that there are no conflicts of interest.

## AUTHOR CONTRIBUTIONS

BQL wrote the manuscript. AS, SS and RKS supervised activity planning and execution. All authors reviewed the manuscript.
